# Academic doctors' views of complementary and alternative medicine (CAM) and its role within the NHS: an exploratory qualitative study

**DOI:** 10.1186/1472-6882-7-17

**Published:** 2007-05-30

**Authors:** Nita Maha, Alison Shaw

**Affiliations:** 1 Torbay Hospital, Lawes Bridge, Torquay, Devon, TQ2 6AA, UK; 2 Academic Unit of Primary Health Care, Department of Community Based Medicine, University of Bristol, 25 Belgrave Road, Bristol, UK

## Abstract

**Background:**

There has been a marked increase in the use of complementary and alternative medicine (CAM) in the UK population in recent years. Surveys of doctors' perspectives on CAM have identified a variety of views and potential information needs. While these are useful for describing the proportions of doctors who hold particular attitudes towards CAM, they are less helpful for understanding why. In addition, while the views of non-academic doctors have begun to be studied, the perspective and rationales of academic doctors remains under-researched. It seems important to investigate the views of those with a research-orientation, given the emphasis on the need for more scientific evidence in recent debates on CAM.

**Methods:**

This exploratory study used qualitative methods to explore academic doctors' views of CAM and the rationales they provided for their views. A purposeful sampling strategy was used to identify doctors with a dual clinical and academic role in the Bristol area, with an anticipated variety of views on CAM. Semi-structured interviews were conducted with nine doctors. The data were analysed thematically, drawing on the Framework Approach.

**Results:**

The doctors expressed a spectrum of views on CAM, falling into three broad groups: the 'enthusiasts', the 'sceptics' and the 'undecided'. Scepticism or uncertainty about the value of CAM was prominent, except among those practising a form of CAM. A variety of rationales underpinned their perspectives on CAM, a key recurring rationale being their perspective on the scientific evidence base. The main themes arising included: the role of doctors' professional experiences of conventional medicine and CAM in shaping their attitudes towards CAM, doctor-patient communication about CAM and patient disclosure, whether there is a need for training and education in CAM for doctors, a hierarchy of acceptability of CAM and the nature of evidence; and the role of CAM within the NHS.

**Conclusion:**

Despite the caution or scepticism towards CAM expressed by doctors in this study, more open doctor-patient communication about CAM may enable doctors' potential concerns about CAM to be addressed, or at least enhance their knowledge of what treatments or therapies their patients are using. Offering CAM to patients may serve to enhance patients' treatment choices and even increase doctors' fulfilment in their practice. However, given the recurring concerns about lack of scientific evidence expressed by the doctors in this study, perceptions of the evidence base may remain a significant barrier to greater integration of CAM within the NHS.

## Background

There has been a marked increase in the use of complementary and alternative medicine (CAM) in the UK population in recent years. CAM has been defined as *:"diagnosis, treatment and/or prevention which complements mainstream medicine by contributing to a common whole, satisfying a demand not met by orthodoxy, or diversifying the conceptual frameworks of medicine" *[1]. For the purposes of this paper, CAM is defined as any treatment or therapy that is not routinely and universally available to patients via the NHS. In 2001, 49% of general practices offered access to some form of CAM, compared to only 39% in 1995 [2]. A recent survey indicated that 10% of adults in England and Wales see a complementary therapist in any 12-month period and 40% have used CAM during their lifetime [3]. Much of this use takes places outside of the NHS, in the form of patients' over-the-counter purchase of CAM treatments (e.g. homeopathic or herbal) or consultations with private complementary therapists. Some authors have warned that patients' growing use of CAM will increasingly impact on 'conventional' NHS consultations, with GPs increasingly having to decide on their attitude towards CAM [4].

Additionally, CAM has noticeably moved up the Department of Health's agenda, particularly in relation to primary care services. This is reflected in the policy document 'Building on the Best: Choice Responsiveness and Equity in the NHS', which included recommendations about working towards a framework for providing patients with access to CAM via the NHS [5]. The advent of practice based commissioning signals a new route by which patients may increasingly access CAM within UK primary care [6].

Quantitative studies of doctors' perspectives on CAM have identified a variety of views and potential information needs. An American survey found that 61% of doctors felt they had inadequate knowledge about the safety and efficacy of CAM therapies and 81% believed that more education was required in this field [7]. Another American survey examined doctors' views of five of the most prominent CAM therapies [8]. They found considerable variations in the perceived effectiveness of the different modalities with approximately half believing that acupuncture, chiropractic and massage were effective, compared with 26% for homeopathy and 13% for herbal approaches.

Some UK surveys of doctors' attitudes towards CAM have been conducted. A survey of GPs in the former Avon area of England in 1986 found that while doctors regarded their knowledge of CAM to be poor, 59% regarded specific therapies (e.g. acupuncture, herbal medicine and homeopathy) to be useful to their patients, particularly favouring spinal manipulation [9]. UK surveys have identified a small but increasing number of GPs who are practising some form of CAM and a growing number of practices that are providing patients with access to certain therapies, most notably through in-house provision [10-13].

However, a review of research on doctors' attitudes to CAM revealed that alongside interest in CAM, doctors raised a number of concerns including safety, lack of proof that therapies work, inadequate knowledge among doctors and absence of statutory regulation for most therapies [10]. Doctors who are not practising CAM may refer to a CAM practitioner in response to patient demand. In a small survey of primary health care workers in Northwest London, 83% had previously referred (or influenced referral) for CAM, the main reason being patient request (68%). There was a significant interest in more training or information (66%) and only 6% were against any integration of CAM into mainstream primary care [14].

While the above surveys are useful for describing the proportions of doctors who hold particular attitudes towards CAM, they are less helpful for understanding why. There is currently little qualitative research examining doctors' perspectives on CAM in more depth, along with the rationales behind their views. A qualitative study of GPs who were personally practising some form of CAM examined their views and experiences of integrating medical and non-medical approaches within their NHS practice [15]. In this study, GPs experienced the practice of CAM as enabling them to engage with both the art and science of medicine, to exercise their clinical autonomy and to resist what they perceived to be the threat of evidence based medicine.

However, there is a need for qualitative investigation of the views of a broader range of doctors, including those who do not practise any CAM, especially regarding whether CAM therapies have a place within the NHS. This is particularly important given recent debates about the integration of CAM within the NHS following the publication of the Smallwood report, [16] which provoked the expression of highly polarised views in the public press.

This exploratory study will begin to address these issues. The aim of this study is to explore academic doctors' views of CAM and its role within the NHS, along with the rationales they give for these views. We chose to focus on the views of academic doctors given the attention to non-academic practitioners' views of different forms of healthcare in previous research [17] and the emphasis on the need for more scientific evidence in recent debates on CAM. Academic doctors might have a particular perspective on the latter, but this as yet remains under-researched.

## Methods

### Study design

Qualitative methods were used as they help us to gain deeper understanding of phenomena from the perspective of participants, giving emphasis to the meanings that participants attach to their experiences and the rationales behind their views [18].

In this applied qualitative study, we adopted a reasonably pragmatic approach, seeking to choose the right methods (semi-structured interviews) to answer our research question ('What are academic doctors' views on CAM and what rationales do they give to support their views?). However, our underlying position is one closely aligned to subtle realist perspectives [19,20]. Underpinning our use of qualitative methods is the belief that the world we research does exist independently of participant's subjective understandings of it, but we can only access it via participant's interpretations, which in turn are interpreted by the researcher. Where different perspectives on reality are expressed by participants, these enrich our understanding of the diverse ways in which reality is experienced, rather than negating that an external reality exists. As researchers, we are aiming to represent a full a picture as possible of that multidimensional reality – in this study, the diversity of academic doctors' views and experiences regarding CAM. However, we recognise that the accounts given to us by participants, and the accounts we construct from these, are "partial" [21], given our limited capacity to fully present the experiences of others.

### Sampling

A purposeful sampling strategy [22] was used to identify a small number of doctors with a dual clinical and academic role in the Bristol area, with an anticipated variety of views on CAM. We approached GPs who had academic links with a local University, from practices with different levels of interest in or provision of CAM. These ranged from GPs from practices with no known interest or provision, through to a practice that rented rooms to complementary therapists, to a practice that offered complementary therapies provided by practice staff, including GPs. The decision was made to focus mainly on GPs, as primary care is the most common NHS setting for any integration of CAM and is where doctor-patient communication about the whole range of patients' treatments is most likely to take place. However, we also included one academic doctor from a secondary care setting, who provided one form of complementary medicine (homeopathy) and received referrals from primary care. Participants were initially identified via personal contacts at known general practices within the local primary care research network, but also through a snowballing method, where interviewees suggested another appropriate person to contact.

### Data collection

**Figure 1:**
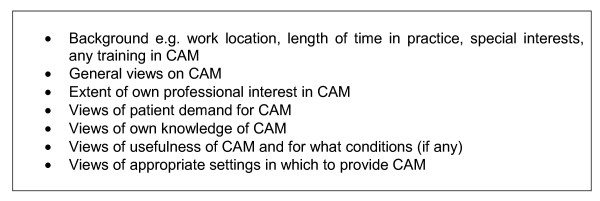
Interview topic Guide.

Data were collected using semi-structured interviews with participating doctors. Interviews were conducted during November 2004. They lasted up to 30 minutes and were held at participants' workplaces. A topic guide was used to ensure that the same broad range of topics was covered with all participants (see Figure 1). However, it included flexibility to allow interviewees to introduce issues of importance to them. All interviews were tape-recorded and the sections relevant to the questions of interest were fully transcribed by the researcher.

### Data analysis

**Figure 2:**
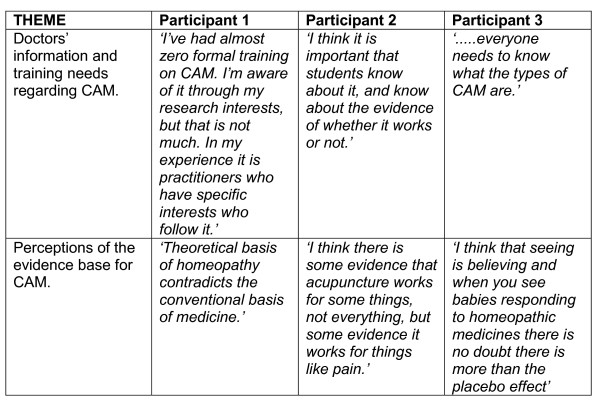
sample analysis matrix.

The qualitative data were analysed thematically, drawing on the Framework Approach, which is often used in applied research where there are pre-identified issues that the researcher wishes to investigate, but with flexibility for new themes to emerge from the interviews and analysis [23]. It involves developing a matrix, where each row is a participant and each column is a theme within the data. Early analysis involved listening to the tapes alongside reading each transcript and noting key issues, with particular attention to those relevant to the study aim. From this, a preliminary code list was drawn up and applied flexibly to each transcript. Next, codes that were similar to each other were merged and a smaller number of over-arching themes were developed that encompassed the majority of codes. The coding and analysis process was led by NM with ongoing input by AS, who read a sub-set of the data, and examined the developing themes and data extracts to ensure there was a good fit. Both authors agreed the final themes to be applied to all interview transcripts. The final set of themes was then mapped across all the individual participants using a matrix, to examine the full range of expressed perspectives on each theme (see figure 2). All data were anonymised for reasons of confidentiality.

The data were coded for both anticipated themes (those arising from the literature, considered in advance and prompted for by the researcher) and emergent themes (those arising spontaneously from participants within interviews). The themes presented in this paper will be illustrated by relevant verbatim quotes reflecting the full spectrum of expressed views.

## Results

Nine doctors were interviewed for this small exploratory study. Eight were general practitioners (GPs) and one was a homeopathic doctor working in a hospital setting and receiving homeopathic referrals from primary care. The latter was included as an 'extreme case' of a conventionally-trained doctor now entirely practising a form of CAM (homeopathy), as a point of comparison with doctors who were practising both CAM and conventional medicine within their general practice or were anticipated to be sceptical about CAM. Such inclusion of disconfirming as well as confirming cases is an important aspect of qualitative research. All GPs had academic links to a local University, in addition to working in various practices.

The key themes from the data to be discussed in this paper are as follows:

• The role of doctors' professional experiences in shaping their views of CAM.

• Doctor-patient communication about CAM and patient disclosure.

• Training and education in CAM: is there a need?

• Hierarchy of acceptability of CAM and the nature of evidence

• The role of CAM within the NHS.

### The role of doctors' professional experiences in shaping their views of CAM

The first theme is the role of doctors' professional experiences in shaping their views of CAM. By professional experience we mean their experiences of practising medicine with their patients. The doctors interviewed expressed a range of perspectives on CAM, and these usually seemed to derive from positive or negative professional experiences of both conventional medicine and CAM. Interviewees fell into three broad categories: at one end of the spectrum were those who were broadly positive about CAM who had undergone further training and practised certain therapies such as homeopathy (the 'enthusiasts'), at the other end of the spectrum were those highly negative about CAM (the 'sceptics') and in the middle those who had not formed a definite opinion (the 'undecided').

Often the doctors who were positive about CAM described specific professional experiences that had influenced them to become interested. Usually these prior experiences took two forms: firstly, positive experiences of CAM and how certain therapies had helped patients; or secondly less positive experiences of conventional medicine, often characterised as a growing dissatisfaction with what they could offer patients within the remit of conventional medicine. Often participants experienced both of the above, which in some instances was life and career-changing, leading them to pursue the practice of particular therapies themselves in order to offer their patients something different to their usual 'mainstream' care. A homeopathic doctor described the underlying philosophies and practice of homeopathy as resonating with a personal need in her as a practitioner, as well as perceiving it to offer a different way to meet patients' needs.

I used to pick up my husband's (homeopathy) textbooks and found them fascinating. They seemed to meet a need in the illness that was not being met in other ways. The literature described human nature in a way that I could recognise; people In a relationship to remedies. It did fulfil a need in me that up until then was lacking. I remember being taken up to a patient and told to 'look at the eye' and the patient had been told 'this doctor will come and look at your eye' and won't look at any other part of you. It was almost like the patient submitted to the process, but I did not think it felt very comfortable... I remember feeling that I wasn't comfortable with the type of medicine I was offering (participant 1, homeopathic doctor working in secondary care)

The doctors practising some form of CAM claimed that this allowed them to practise medicine in a more holistic way, allowing the patients more voice to express their stories and enabling the doctor to begin to get beneath surface symptoms. The extra time that CAM consultations allowed was viewed as a vital part of this, providing the opportunity for fuller exploration of issues important to patients that time constraints in routine general practice consultations did not allow. A GP who practised homeopathy suggested that this enabled him to enhance the conventional care he provided to patients. He went on to illustrate this by giving an example of how a homeopathic consultation provided an alternative space for discussing in-depth the reasons behind a patient not wanting surgery, giving the GP time to work with the patient to accept it on their own terms.

*(It is a) refreshingly different approach with a much more positive involvement of the doctor in the healing process...We have an hour with the patient and believe me that is not enough either... Time allows the patient to tell their story, and I don't think that five minutes in a quick GP appointment ever does more than hit the surface symptoms and protects the doctor from getting involved (participant 2, GP, trained in CAM)*.

If we [doctors] can understand the reason why they [the patients] don't want surgery, such as the fear of the knife or an experience they've had before, we can work with them to accept surgery on their terms...swe can bring them round to accepting surgery (participant 2, GP, trained in CAM)

In the accounts of doctors who expressed more negative views about CAM, there were a variety of underlying reasons. A recurrent expressed concern was that complementary or alternative therapies could give patients false hopes of cure. Doctors distinguished between therapies that claimed to offer a 'cure' and those that offered supportive care to improve patients' sense of wellbeing, seeming to feel more comfortable with the latter.

If they [CAM therapies] are sold to the patient as a cure for a condition, then I am very wary. If they are sold as something that may help them, make them feel a bit better...than I am happy with that (participant 3, GP, not trained in CAM)

Apparent within these concerns about false cures were protective attitudes on the part of the doctors towards their patients, which seemed to be fuelled by suspicion of financial 'scams' by 'unscrupulous' complementary therapists. This was particularly so for conditions where patients were perceived as especially vulnerable, such as cancer. Doctors sometimes provided specific examples to illustrate their concerns. An example was a GP who described how one of his patients with cancer had paid a large amount of money for a brief telephone consultation and treatment of "no proven value".

Doctors who were undecided about CAM often claimed that they lacked sufficient knowledge of CAM to make an informed judgement. They also drew on arguments about the uncertainty of scientific evidence regarding CAM. They acknowledged that some research evidence indicated that certain CAM therapies might be effective or helpful, cautiously accepting that these might benefit certain patients. Where doctors had direct experience of taking part in research evaluating a CAM therapy, this prompted interest and potential support for the therapy, particularly when the study results looked favourable. However, caution was predominant, as the 'undecided' doctors argued that further research was needed to confirm for certain any beneficial effects, and wanted further scientific evidence on other less studied therapies or therapies where the evidence was more equivocal. This was particularly the case for therapies where they found it hard to understand *how *it worked, such as homeopathy.

*I am undecided on a lot. I don't know much about CAM if I am honest. I am aware of some particular types of CAM, e.g. we had a research project In our practice looking at the Alexander technique for back pain and I am quite intrigued by that...sit looks like a goer. Homeopathy, what is homeopathy? There is some evidence that it works, but my question is how? (participant 8, GP, not trained in CAM)*.

### Doctor-patient communication about CAM and patient disclosure

The second broad theme is doctor-patient communication about CAM and patient disclosure of CAM use. Discussions with patients about CAM initiated by the doctor were reported to be unusual among the GPs interviewed, particularly among those who were sceptical towards or undecided about CAM. Most acknowledged that they would only discuss CAM when a patient raised it within a consultation. The reason for this was that they did not feel that CAM should be a priority within the consultation when the scientific evidence was not strong.

Where GPs did initiate a discussion about CAM, some acknowledged that this would often only be as a check – "to see if they are using anything" – rather than as a positive encouragement to try a CAM therapy. This was particularly so among those who were "undecided" about or sceptical towards CAM.

*I discuss the subject of [CAM] to see if they are using anything, but I don't encourage them to use it (participant 5, GP, not trained in CAM)*.

Among the minority of GPs who initiated the subject, including those who practised a form of CAM, there was acknowledgement that this often took place at a later stage of the consultation, after conventional options had first been discussed. Thus within primary care consultations, CAM was not usually a first port-of-call but a backup when conventional approaches had little to offer the patient. The reasons for offering CAM to patients included being able to offer patients something that would "do no harm" as an alternative to pharmaceutical medicines that may have unpleasant side effects. Patient choice seemed to be an influential factor for some doctors. For example, a GP practising CAM claimed that he might be more likely to suggest CAM when he is dealing with patients who are not "typical" CAM users, in order to offer them "another option" that the patient is unlikely to have considered. Patient preferences were also referred to as a reason for considering CAM rather than conventional approaches. For example, one GP mentioned that he had discussed St John's Wort with a patient who was suffering from depression, when the patient stated he was strongly against using anti-depressants. However, patient preference alone might not be sufficient, as this GP's decision to suggest St John's Wort was at least partly based on the perception that there was "some evidence" that it worked.

*I tend to bring up the subject [complementary therapies] myself because our patients aren't aware of it most of the time. They aren't your typical complementary medicine user...they won't have friends or family who use it. It's another option...for me to suggest something that may help them and won't do any harm is quite a novel experience. Most doctors don't do something like that, they give them horrible pills which make them feel sick, and make them have blood tests (participant 3, GP, not trained in CAM)*.

I had one patient with depression who really didn't want to take any antidepressants. I suggested that they tried taking St John's Wort as I heard this could work for depression, and there is some evidence for it (participant 2, GP, trained in CAM)

Among those doctors who were sceptical towards CAM, the usual cited reason for not raising or recommending CAM within consultations was belief in a lack of scientific evidence of effectiveness. All of the doctors claimed that they would support patient choice by being willing to refer patients to complementary therapists if they requested it. But aside from the CAM 'enthusiasts', they would not initiate such a referral.

*I would consider referring patients [to complementary therapists] if they requested it. However, I would have to emphasise that there is little evidence (participant 6, GP, not trained in CAM)*.

Those who were sceptical or uncertain about the value of CAM acknowledged that their patients might be reluctant to disclose their CAM use, or request referral to a CAM practitioner, if they perceived the doctor's scepticism. Thus, they recognised that the extent of doctor-patient communication about CAM within consultations is likely to vary depending on the doctor consulted and the degree to which they reveal their personal attitudes towards CAM to their patients. This is turn is likely impact upon how much the doctor knows about the patients' own treatment choices and self-care with CAM outside of their conventional NHS care.

*None of my patients have ever asked me if they could see a homeopath – maybe because they pick up my scepticism! (participant 5, GP, not trained in CAM)*.

### Training and education in CAM: is there a need?

The third broad theme concerns whether there is a need for doctors to undertake training or education in CAM. The participants were broadly aware of a variety of training options for doctors in specific CAM therapies, the most commonly cited being homeopathy, acupuncture and nutritional medicine (for general practitioners in particular). When questioned about any experiences of training in CAM, most stated that they had no formal tuition, and the minority who had qualifications in specific therapies (typically homeopathy) had proactively sought the training as a result of a personal desire to practise that therapy. Rather than detailed knowledge, the majority had general awareness of CAM, which was often gained through their research interests, rather than their clinical practice. There was recognition that only those with particular interests in CAM seeking training.

*I've had almost zero formal training on [CAM]. I'm aware of it through my research interests, but that is not much. In my experience it is practitioners who have specific interests who follow it (participant 6, GP, not trained in CAM)*.

Those with no training in CAM expressed varying degrees of interest in and willingness to undertake training on CAM. Among those open to receiving some training (usually the 'undecided'), they suggested that what would be acceptable was a short introductory course that provided a brief overview of each therapy to enhance their general awareness, rather than in-depth training in particular therapies. However, training in CAM competed with other priorities, and in such competition was unlikely to win, particularly given the time constraints within practice that limited their opportunities for training. A minority (the 'sceptics') had no interest in learning more about CAM. This was largely due to a lack of interest in a subject that they perceived as having no real evidence base.

It's a case of thinking that if I go on that course I can't do something else. I wouldn't mind having a day on the different types of [complementary therapies] available – a summary, the philosophy, the training, the kinds of people they see, preferably any evidence of benefit or harm...there's a huge number of therapies..I have a vision of a half an hour talk on each (participant 3, GP, not trained in CAM)

I would consider having more training on it, but it is not a priority for me; I have other interests. I would consider it if there was a stronger evidence base (participant 8, GP, not trained in CAM)

Despite mixed views about the value of professional development in CAM once qualified, there was consensus about the need to include CAM within the undergraduate medical curriculum, although views on the extent of inclusion varied. One cited benefit of including CAM was that it could broaden medical education, by encouraging students to be holistic practitioners and understand the art, as well as the technical aspects, of medicine. The majority of the doctors did not feel that detailed teaching was necessary, due to the perceived small numbers of students who would go on to practice CAM and who would therefore need in-depth knowledge of the subject. As a minimum, they thought that future doctors should be generally aware of the types of therapies available and understand the evidence base, particularly whether particular therapies "work".

In undergraduate medicine we want to get across holistic medicine. All doctors should be holistic, humanistic doctors who understand the art of medicine, not just the technical things. Only a small minority in the year will practice [CAM therapies]. On the other hand, everyone needs to know what types of [CAM therapies] there are (participant 7, GP, trained in CAM)

However, greater introduction of CAM into the undergraduate medical curricula was seen to raise some tensions. Most notably, because of a perceived lack of evidence of effectiveness of CAM therapies, there were concerns that including teaching on CAM could contradict the principles of evidence-based medicine that underpin the curricula. In contrast, this very tension was cited as a virtue by one GP. He argued that a benefit of teaching about CAM was that it could raise awareness among students about the uncertainty and limits of medical knowledge. This was contrasted with the perceived certainty of complementary therapists that they can help their patients, which was criticised.

I think it is important that students know about it, and know about the evidence of whether it works or no. I am concerned that one day students would be taught about evidence based medicine, and then the next day they would be taught in a completely different mind view by someone not very interested in it (participant 5, GP, not trained in CAM)

It is extremely important in the undergraduate curriculum for students to acknowledge when they don't know the answers...It is extraordinary how alternative medicine practitioners are so certain they can help everyone (participant 9, GP, not trained in CAM)

### Hierarchy of acceptability of CAM and the nature of evidence

The fourth theme concerns a hierarchy of acceptability of different CAM therapies, which is underpinned by views about the nature of evidence. As already seen throughout the previous themes, ideas about 'evidence' underpinned much of the doctors' accounts, forming a core component of the rationales for their views of CAM. The majority of the doctors were not convinced by the current scientific evidence for CAM and said that they wanted further research to establish the effectiveness of specific modalities, emphasising the importance of collecting evidence that could withstand strong critique, most notably, the 'gold standard' of randomised controlled trials.

Those who were practising a form of CAM (notably homeopathy) also expressed uncertainty about the levels of 'scientific evidence' and acknowledged how they grappled with the need for scientific explanation of how a treatment or therapy works, particularly early in their practice of CAM. However, these doctors often drew on other forms of 'evidence' to support their understanding that a therapy worked – primarily, repeated professional experience of witnessing benefit to patients. For those practising homeopathy, the most significant factor convincing them of its power to heal was witnessing its effect first hand in their patients. This was described as "seeing is believing". This experiential evidence countered the scientific critiques of homeopathy and caused them to focus less on fully understanding the precise mechanism of action, enabling them to accept that something can work even if it doesn't have a scientific explanation. They suggested that "ignorance" was the greatest barrier to other doctors accepting homeopathy.

Ignorance... I still site that as the main reason why my colleagues and the doctors I work with think it [homeopathy] is a load of hocum pocum..... our greatest problem is that there is no scientific explanation and I used to get very hung up on the concept of dilutions and potencies and how can something with nothing in it have any effect? But once I witnessed more and more the effect in clinical practice, I became a lot less hung up on the mechanism of healing........ I think that seeing is believing and when you see animals and babies responding to homeopathic treatment there is absolutely no doubt that there is more than the placebo effect..... how can a baby respond to that [the placebo effect] when you can't explain that in psychotherapeutics (participant 2, GP, trained in CAM)

The doctors who were most sceptical about CAM held strongly to the need for scientific proof and drew on ideas about rationality. They argued that CAM treatments are a 'flight from rational thought' because belief in the benefits of therapies cannot be reconciled with the lack of scientific proof. There was a suggestion that they only 'work' and are popular with certain patients because they are marketed as alternatives to pharmaceutical medicines, which some patients are averse to taking.

I think that most research shows it doesn't work and yet people continue to believe in it, and that reinforces my view that it is a flight from science and rational thought......some bits of complementary medicine don't want to become mainstream. The whole attraction is not to be mainstream. If a drug company marketed homeopathic medicines they would stop working. I think it is all to do with the fact that it's not made by drug companies (participant 5, GP, not trained in CAM)

The doctors differentiated between forms of CAM, identifying those they felt comfortable with and others they were less confident in. Thus, a hierarchy of acceptability of CAM therapies emerged, reflecting the diversity of therapies available, and doctors' varying beliefs about the problems and merits of particular modalities. The majority of participants offered a qualified view that some research studies had shown that certain types of CAM could be effective for particular conditions or patient groups, for example acupuncture for pain. But there was some concern over the generalisability of these findings, with caution expressed regarding whether a therapy works for a broader spectrum of conditions and whether treatments effective for one individual would necessarily be effective for another. Modalities perceived as most effective were generally more 'mainstream' treatments such as medical acupuncture, those seen to have pharmacological basis similar to conventional medicine (for example, certain herbal medicines) and those perceived to be helpful for particular conditions (for example, acupuncture for pain). Other forms of CAM were perceived as "nice" in terms of enhancing patients' wellbeing, and the doctors were less concerned about the level of scientific evidence for such, as they were seen to be non-invasive, harmless and potentially enjoyable for patients (for example, aromatherapy massage).

I think there is some evidence that acupuncture works for some things, I don't think that it works for everything, but there is some evidence that it works for some things like pain (participant 6, GP, not trained in CAM)

Therapies I feel more comfortable with are osteopathy, chiropractic and acupuncture. I don't understand or believe their physiological basis because it doesn't correlate with what I have been taught in the dissecting room or physiology lab, but there seems to be a fair amount of evidence that these sorts of treatments seem to be effective...there are others which are just a nice, good thing...aromatherapy massage...sI know they are a good thing... then there are others which go against their rationale...so called healing, which I am wary about (participant 3, GP, not trained in CAM)

Homeopathy was the subject of particular debate. The homeopathic concept of dilutions was often met with cynicism, with several of the doctors questioning how diluted agents could still be pharmacologically active. Those sceptical about homeopathy were critical of publishing processes for homeopathic research, arguing that only positive studies of homeopathy tend to be published, which they saw as a barrier to gaining 'balanced evidence'. Those who practised homeopathy argued that whilst counter-intuitive to those trained in conventional medicine, there could be a particular mechanism of action that is yet poorly understood and noted that further research is required to gain insight into this.

The theoretical basis of homeopathy contradicts the conventional basis of medicine. The concept of treating potency with increasing dilutions is alien to me (participant 4, GP, not trained in CAM)

*I think homeopathy is the one with the most studies done. Studies that were placebo controlled showed that it did work. The problem with this is the positive studies get published and the negative ones don't. Therefore getting a balanced view is difficult (participant 6, GP, not trained in CAM)*.

*Although it (homeopathy) is counterintuitive in a pharmacological basis, it might not be at a molecular level. It's an interesting and developing field. We need to do lots more research (participant 3, GP, trained in CAM)*.

### The role of CAM within the NHS

The fifth and final theme concerns doctors' views about the potential role of CAM within the NHS. There were two broad groups of participants: those who thought that CAM required greater priority within the NHS and those who were against further integration. Costs were a key issue for both groups, but their views on the costs of incorporating more CAM within the NHS and the rationales for their views varied. Participants in the former group believed that incorporating CAM into the NHS could potentially be a cost saving measure. They argued that whilst CAM consultations may be longer and therefore more expensive, they could have the potential to save costs in the long term through reducing repeat consultations over a period of time and minimising the use of expensive conventional treatments.

What a lot of doctors and health economists don't realise is that although homeopathy may take longer with the patient, they come back less often as a result. The remedies are very cheap, and it is only 2 or 3 tablets. Not packet after packet month after month, year after year. So it may take time but in the long run the time has actually been shown to be cut down by repeat visits (participant 3, GP, trained in CAM)

Doctors in the latter category took a different approach to resource issues, arguing that increased NHS spending on CAM would inevitably mean reducing spending on other areas of healthcare given the finite and already highly stretched NHS budget, which was not perceived as favourable.

When you ask should something be funded, you have to ask what should you not fund to pay for this? (participant 5, GP, not trained in CAM)

Views about scientific evidence were once more a core part of participants' rationales regarding integration of CAM within NHS services, particularly among doctors who were sceptical about the place of CAM within the NHS. Without improved evidence, greater NHS funding for CAM services was seen as undesirable or unlikely. "More research" was a common call. Without more research that definitively establishes effectiveness, they argued that the place of particular CAM modalities within the NHS, and the level of funding certain therapies should receive (if any), could not be established.

I think [CAM therapies are] a low funding priority until the evidence base is better. I think with an improved evidence base it would crawl up the funding ladder (participant 5, GP, not trained in CAM)

## Discussion

This study has provided a qualitative account of academic doctors' views of CAM and the rationales underpinning their perspectives. While this is a small exploratory study, the findings indicate a spectrum of views on CAM. Key themes include the role of doctors' professional experiences of conventional medicine and CAM in shaping their attitudes towards CAM, doctor-patient communication about CAM and patient disclosure, whether there is a need for training and education in CAM for doctors, a hierarchy of acceptability of CAM and the nature of evidence; and the role of CAM within the NHS.

Professional experience played a key role in shaping doctors' perspectives. Those who were positive about CAM were usually motivated by encouraging experiences of how CAM had helped patients and for some this had led them to undertake training in CAM. In terms of the impact of CAM on their own practice, aspects that doctors practising CAM particularly enjoyed included the holistic approach and more time with patients, which contrasted with the constraints of routine consultations. This supports the suggestion of Owen *et al *that one of the main reasons why doctors undertake training in CAM is to divert away from conventional medicine, which is often stressful and can be limited in what it can offer patients [24].

The main rationale amongst those doctors who were negative about CAM was the perceived absence of a strong evidence base, which echoes wider views expressed by health professionals in debates about CAM [25]. They were also concerned to protect their patients from what they perceived to be potentially unscrupulous complementary therapists and expensive "unproven" therapies, particularly "vulnerable" patients such as those with cancer.

A hierarchy of acceptability of different CAM therapies emerged from the doctors' accounts. Those endorsed were those most closely aligned with conventional medicine, that were seen to have greater evidence of benefit for certain conditions, notably acupuncture, osteopathy and chiropractic. These findings concur with those from questionnaire studies, that the majority of GPs endorse the use of a limited range of more mainstream therapies, notably acupuncture, chiropractic and osteopathy [26]. Homeopathy remained controversial, with participants expressing difficulty accepting its theoretical basis and mechanism of action.

The GPs in this study reported that where CAM is discussed in consultations this is usually initiated by patients. However, there was some acknowledgement that patients may be unlikely to raise the topic if they perceive the doctor to be sceptical. This mirrors research with patients by Stephenson *et al*, which found that patients might not raise their self-care practices, including CAM use, within consultations because of concerns about their doctor's response [27]. While the doctors in this study reported that discussion of CAM was primarily initiated by their patients, a minority suggested CAM therapies to their patients, for example a GP in a more deprived area where a subsidised CAM service existed within the practice which patients may not have been aware of. Thus, there may be particular circumstances where doctors initiate a discussion of CAM within consultations, primarily if they have a personal interest in CAM, if they wish to enhance patient choice, or if there is a trusted CAM service within or near the practice that is affordable to patients.

There was some recognition amongst participants that the numbers of patients requesting CAM is increasing, which supports the findings of larger prevalence studies noting growth of public use of CAM [28]. Some authors have argued that, despite the potentially limited evidence base, increased patient demand should be a key driver for greater integration of CAM within conventional medicine, to address patients' needs and preferences [24].

Regarding training and education issues, there was consensus among the doctors in this study that the inclusion of CAM in the undergraduate medical curriculum was acceptable and necessary, with some provisos. Concerns rested mainly on fears about undermining the principles of evidence based medicine through teaching about "unproven" therapies. CAM has begun to be included in undergraduate curricula in some Universities, often in the form of optional special study modules [24]. Inclusion of training in CAM at undergraduate level may be more fruitful than at post-qualifying, as Zollman and Vickers found that older general practitioners tended to be the most sceptical about CAM, with medical students and younger doctors more interested [29]. Since enthusiasm for CAM amongst patients is expanding, and patients will increasingly be seeking information about CAM from health service providers, the need for doctors to have enhanced awareness, if not detailed knowledge, of CAM is likely to grow.

Scepticism about the scientific evidence base for CAM was a strong feature of doctors' accounts. This was a key factor in their views and decisions on many issues, such as whether they would discuss CAM with patients in consultations, whether they saw a need for further training in CAM and whether CAM had a place within the NHS. Those doctors practising a form of CAM argued against what they saw to be a narrow model of evidence, calling for different forms of evidence of patient benefit, namely patient experience. Such evidence had been powerful in the lives of these doctors, leading them to train in a form of CAM in the first place, or confirming to them the validity of the therapy once practised.

Doctors' polarised views about the place of CAM within the NHS reflected recent wider debates in the media about integration. For example, the Smallwood Report brought controversy with its suggestion that complementary therapies provided on the NHS could potentially result in a long-term reduction in treatment costs [16]. Some doctors who were concerned about the increasing public profile of CAM and the findings of the Smallwood Report expressed their reservations in a letter to the Times newspaper, outlining the need for effective distribution of resources on treatments of proven benefit. They argued for the importance of reliable evidence on the cost-effectiveness of CAM before any therapy is provided within the NHS [30].

While there were familiar calls for more research in this study, particularly among the 'sceptics', the relative paucity of research on CAM to date is due historically to lack of research funding. Vickers has suggested that an increased amount of good quality CAM research is being conducted worldwide, reflecting growing awareness of its importance amongst researchers [31]. Yet, it is worth noting that research funding for CAM still lags significantly behind that for conventional medicine [32].

### Study limitations

Our participants expressed a range of views on CAM and its role within the NHS. However, this was an exploratory study and only a small number of academic doctors were interviewed. The ideal in qualitative research is to continue sampling and collecting data until data saturation is reached. In this small-scale study, practical constraints limited the time and resources available for gathering and analysing data. We acknowledge that the study themes merit further examination in a larger qualitative study with a broader sample of doctors from a more diverse range of settings, both community and hospital based. While participants were given a copy of the final findings, we also recognise that it might have been useful to assess the credibility of the themes and our interpretation of them at earlier stages of the analysis process, through strategies such as respondent validation. Our findings may have some transferability to academic doctors in other similar primary care settings, with or without a professional interest in CAM. However, as all of our participants had an academic role, their views are unlikely to be typical of non-academic doctors. Ideas about scientific evidence were a prominent thread throughout their accounts, which may be a reflection of the research orientation of the particular doctors sampled. Therefore, the findings of our study may over-emphasise the importance of scientific evidence to doctors, as a previous study of the views of healthcare providers in a non-academic setting found little reference to research and evidence as important factors determining the acceptance of CAM within mainstream healthcare systems [17]. Furthermore, given the polarised views of CAM expressed in this study, we may have under-represented the views of more "middle ground" doctors, which could be accessed through a future broader sample incorporating non-academic doctors. Future sampling criterion could also include other individual GP and practice factors that may influence doctors' views, such as: length of time since training/in practice; practice location (e.g. urban/rural); and socio-demographics of the patient population. It would also be interesting to include doctors with or without direct personal experience of using CAM as a patient, as this is likely to shape their perspectives on CAM use for their patients within their professional role.

## Conclusion

This exploratory qualitative study has examined the range of perspectives on CAM amongst academic doctors and the rationales they provide for their views. Scepticism or uncertainty about the value of CAM was prominent, except among those practising a form of CAM. However, despite this caution or scepticism, it seems important that doctors facilitate an atmosphere of openness within consultations, so that interested patients feel able to discuss CAM, particularly in an era of patient-centred medicine where choice is a key watchword. More open doctor-patient communication about CAM may enable doctors' potential concerns about CAM to be addressed, or at least enhance doctors' knowledge of what treatments or therapies their patients are using. Offering CAM to patients may serve to enhance patients' care options and even increase doctors' fulfilment in their practice. However, given the recurring concerns about lack of scientific evidence expressed by the doctors in this study, perceptions of the evidence base may remain a significant barrier to greater integration of CAM within the NHS.

## Competing interests

The author(s) declare that they have no competing interests.

## Authors' contributions

Both authors contributed to the design, analysis and writing-up of this study. NM was responsible for data collection and producing the first draft of this paper.

## Pre-publication history

The pre-publication history for this paper can be accessed here:


